# News and Notes

**Published:** 1906-04

**Authors:** 


### NEWS AND NOTES.

NATCHEZ, MISS., has passed an anti-mosquito ordinance as a preventive of yellow fever.

THE plague situation in Japan seems to be ameliorating, although the disease has not been entirely suppressed.

IN Illinois last year there were 65,000 deaths and 150,000 births. The males born exceeded the females by about 6,000.

DR. J. R. RATHMELL has been elected Dean of Chattanooga Medical College, to fill the place of Dr. E. A. Cobleigh, deceased.

THE Medical Society of the State of New York celebrated its centenary by a banquet at Odd Fellows' Hall, Albany, January 31. A centennial history will soon be issued.

THE Ohio Senate has passed a bill abolishing capital punishment for persons convicted of murder in the first degree, except when the conviction shall be for a second offense.

THE Yerkes hospital foundation of six million dollars was established by the late Charles Tyson Yerkes. A great hospital will be built in the Bronx Borough out of this benefaction.

UNDER the auspices of the Kentucky Club, of Denver, Colo., a tuberculosis sanitorium is to be founded near Denver for the reception of tuberculous natives of Kentucky, who will receive treatment at low cost.

IT is reported, by way of Berlin, that the Russian cholera commission has declared the Vistula district, the districts of Wolhynia and Kurland, and the city of Lomza free from cholera.

---

AT a meeting of the council of the Michigan State Medical Society, held in Detroit on January 12, 1906, Dr. A. P. Biddle resigned, and Dr. B. R. Schenck was elected State secretary and editor of the journal.

---

A NEW medical college founded by a union of four Protestant missions and established with the imperial approval was to be opened at Peking on February 1, 1906. It will be known as the Lockhart Medical College.

---

AT a meeting of the board of trial of the Massachusetts Medical Society, held January 31, Dr. Percy D. McLeod was found guilty of unprofessional conduct. The penalty for this offense is expulsion from the society.

---

A CHAIR of social medicine has been founded in the University of Bonn. It has been accepted by Dr. Rumpf, professor of internal medicine and some time director of the medical section of the general hospital at Hamburg.

---

AN ordinance to prohibit the free distribution of samples of patent medicines, drugs, ointments, pills, powders and pellets on the streets or from house to house in the city of Grand Rapids, was passed by the common council, February 19.

---

IN the States of the German Empire there is a tariff regulating the charges to be made by the physician giving a high and a low mark. To this has been added lately the following remark:

"Physician's advice by telephone is to be charged as a visit to the office."

---

DR. J. B. MATHEWS, of Greensboro, N. C., charged with murder of his wife, by injection of strychnine while she was ill, and while he was pretending to be offering a prayer at her bedside, was declared guilty and has been sentenced to the penitentiary for twenty years.

---

MAX NITZE, M.D., professor of urology at Berlin, and inventor of the cystoscope, died suddenly, February 22, aged 58. The twenty-fifth anniversary of this invention was celebrated in March, 1904, when honors were showered on Nitze, who was then only a *privat docent*.

---

DR. J. W. GUSTIN, chairman of the Bay City, Mich., board of health, has located the source of the trichinosis infection which has caused more than thirty cases of illness and one death in the city, at a farm near Vassar, where the hog was purchased from which the infected meat was cut.

---

THE captain of the steamship Erny of the Austro-American Line has been arrested, charged with providing unwholesome food for infants in the steerage. Several other captains of ships in the immigrant trade have also been arrested for failing to obey the statutes relating to accommodations for steerage passengers.

---

THE physicians of Taliaferro county, Ga., at a recent meeting, at Crawfordville, organized a county medical association, with the following officers: Dr. Arthur C. Davidson, president; Dr. John A. Rhodes, vice-president; Dr. A. H. Beazley, secretary; Drs. Lawrence R. Brown, Power and John A. Rhodes, censors.

PRESIDENT ROOSEVELT has appointed a joint board, composed of officers of the medical department of the army and navy, to consider improvements in the matter of first-aid dressings, and the advisability of the adoption of a uniform equipment in the medical department of the two principal branches of the military service.

---

THE Berlin Royal Museum has acquired a large collection of recently discovered medical papyri, which will be published in due course. The contents, which are of the most varied description, give striking proof of the activity of medical thought in ancient Egypt. Among the papyri are two on which letters from Hippocrates are inscribed.

---

THERE seems to be hope of being able to utilize atmospheric nitrogen in commerce, and Norway is producing 1,200 pounds of nitric acid annually from this source. The vital feature of the enterprise is the union of atmospheric nitrogen and oxygen by means of heat from a magnified electric spark. The idea is fascinating, and with inexpensive power may become practical.

---

FOR the first live female stegomyia found in any house in New Orleans and delivered to the city board of health a reward of five dollars will be given; the same amount will be paid for the second and two dollars for the third. Then there is a reward which applies to the entire family of stegomyia. Five cents will be paid for each stegomyia, dead or alive, female or male, young or old, found in any house within the Crescent City.

---

DUKE CARL THEODORE, of Bavaria, is a well-known physician, having long made ophthalmology his specialty. He recently performed his five-thousandth cataract operation, assisted by Dr.

Zenker, and by his wife, who was the Princess Maria, of Portugal. She has always taken an interest in his work, and frequently assists him. It is said that he never accepts a fee from any patient, devoting his services to the poor. He lives in Munich, and has made many endowments for hospitals and other charitable purposes.

---

THE board of health of the City of Mexico has begun a campaign against insanitary conditions. It will require that premises be kept clean, and that landlords furnish a larger supply of water in the tenement houses. Bathhouses will be built at all police stations, and dirty criminals will be compelled to bathe. The dirty clothing of the very poor will be burned, and new raiment will be provided. Congested districts are to be dispersed. As one would judge, the inspectors of the health boards are to be invested with extraordinary powers.

---

DR. L. DUNCAN BULKLEY gave two special lectures on the Principles and Application of Local Treatment of Diseases of the Skin, March 21 and 28, and will deliver two more on the same subject April 4 and 11, at the New York Skin and Cancer Hospital.

Dr. William Seaman Bainbridge will also give a clinical lecture on Malignant and Non-malignant Growths, on April 18, in the out-patient hall of the hospital.

These lectures are free to the medical profession.

---

INTEREST is increasing in the approaching session of the International Congress, which is to be held at Lisbon, April 19 to 26. John H. Musser, of Philadelphia, is chairman of the National Committee, and Ramon Guiteras, of New York City, is the secretary, to whom all applications for membership in the Congress and communications regarding papers should be addressed. The sailing date of the American party is April 7, by the North German Lloyd steamship Konig Albert. The arrangements are in the hands of Charles Wood Fassett, of St. Joseph, Mo., to whom applications for reservations should be made.

---

THE American International Congress on Tuberculosis is hereby called to meet in New York City, November 14, 1906. A joint session of the Congress and the Medico-Legal Society will be held. Titles of papers and the names of members who will attend should be sent to the secretary at as early date as possible. Full particulars will be published in the medical and lay press next month. By order of the EXECUTIVE COMMITTEE.

Approved:

F. E. DANIEL, M.D., President, Austin, Texas.

M. M. SMITH, General Secretary, Austin, Texas.

---

DR. IRA REMSEN, of Johns Hopkins, as the guest of honor and principal speaker at the annual dinner of the Alumni of Johns Hopkins University, in speaking of Dr. Wm. Osler and the "old age theory," said: "Professor Osler is a very sensitive man, and the notoriety he has gained unwittingly is very painful to him. When he went some time ago to Atlantic City for a rest, which he so much needed, he was obliged to travel incognito, and at the hotel where he stayed he did not sign 'William Osler,' but used some other name. Again, when he went to England he went under another name to avoid questioning as to his theory."

---

ACCORDING to the London *Express,* the entire staff of medical men attached to the Exeter Dispensary have decided to strike. Insults, they declare, have been heaped on them, and "the profession of medicine degraded by the base calumnies of the committee." The trouble has arisen from a proposal by the committee to appoint a paid medical officer. In 1886 the medical staffs of the hospitals of Rome decided to strike on a certain day. When the day arrived each physician received from the ministry of war a commission in the army, with orders to report for service to general officers who had simultaneously been appointed to take charge of the various hospitals. In these circumstances a strike would have been "mutiny." There was no mutiny among the new members of the Italian army's medical staff.—*Medical Age.*

---

THE Terrell County Medical Society met in session February 6, and the following officers were elected for the ensuing year: Dr. J. G. Dean, of Dawson, Ga., president; Dr. O. G. Cranford, Sasser, Ga., vice-president; Dr. H. W. Harris, Dawson, Ga., secretary and treasurer.

Delegate to meeting of Georgia Medical Association in Augusta, Dr. W. B. Cheatham, of Dawson. Dr. H. W. Harris, of Dawson, alternate. Board of Censors: Dr. O. G. Cranford, Sasser; Dr. Guy Chappell, Dawson, and Dr. J. T. Arnold, Parrott.

The Society has enrolled every physician of the county, and all are enthusiastic and hopeful of the good that may be accomplished along the different lines in this vicinity. All meetings are held at the court-house in Dawson, Ga.

---

AUTOPSIES made at the Bellevue morgue show that some deaths have been due to fine particles of steel in the lungs. This statement was made public after autopsies made on two subway track walkers within the past month. Dr. Soper, in his report to the rapid transit commission, declared that in samples of subway dust examined by him there occurred more than 60 per cent. of the minute steel particles, and it has been established that the steel dust generated by friction in the subway amounts to a ton a mile each month. Dr. Soper finds the subway cleaner than the streets. General results show that there are twice as many bacteria in the air of the streets as there are in that of the subway. The subway could be made more healthful by substituting a smooth concrete surface for the broken stone ballast between the tracks that forms a nesting place for dirt and germs.—*Journal Am. A., March* 10, 1906.

---

AT the meetings of most medical societies the demonstration of patients and specimens is secondary in importance to the discourses. When too much attention is devoted to them, the scientific papers are belated. A number of physicians in Berlin have organized an informal society for demonstrations only, each demonstrator being limited to ten or fifteen minutes and only five minutes being allowed for discussion at the time. When it is desired to have discussions exceed this limit, they must be transferred to the arena of the already established medical societies. The first meeting of the new organization was held in January. Among others, Brieger exhibited specimens of syphilitic lesions of the liver, simulating tumors, while Karewski presented some patients with these lesions, incorrectly diagnosed at first. Albu exhibited cases of stenosis of the pylorus and Pinkus demonstrated the *Spirochœta pallida.* The society bears the euphonious name of the "Zwanglose Demonstrationsgesellschaft." The description in the *Allg. Med. Ct.-Ztg.* states that the meetings are held at the amphitheater, Ziegelstrasse 18.—*Journal Am. A., March* 17, 1906.

---

A PERSISTENT, chronic discharge from the nose should lead one to suspect chronic disease of the frontal or other accessory sinus. —*American Journal of Surgery.*

